# Lipid parameters in a hyperendemic area for malaria

**DOI:** 10.1186/1476-511X-12-162

**Published:** 2013-11-01

**Authors:** Frederico AR Neves, Ana MRS Ventura, Manoel GS Filho, Rosana MF Libonati

**Affiliations:** 1 Instituto Evandro Chagas, Rodovia BR 316 – Km 07 S/Nº, Levilândia, Ananindeua, PA 67030-000, Brazil; 2 Núcleo de Medicina Tropical, Universidade Federal do Pará, Av. Generalíssimo Deodoro, 92, Nazaré, Belém, PA 66055-240, Brazil

**Keywords:** Malaria, Cholesterol, *Plasmodium*

## Abstract

**Background:**

This is a cross-sectional study with the objective to analyze lipid parameters of individuals living in Brazilian Amazon, where malaria is endemic.

**Methods:**

The city chosen was Anajás in the state of Pará, Brazil, in Amazon region. The study analyzed lipid parameters of 46 subjects, 31 male and 15 female, aged between 20–60 years without malaria, and residents for more than five years in this city considered an area hyperendemic for disease. It was established three groups according to the number of previous episodes of malaria: group I (n = 22) one to five episodes, group II (n = 20) six to ten episodes and group III (n = 4) eleven to fifteen episodes. Total cholesterol, high density lipoprotein (HDL cholesterol), and low density lipoprotein (LDL cholesterol) were measured and was confected the thick smear for malaria of all individuals.

**Results:**

The hypocholesterolemia, the main characteristic of hyperendemic areas for malaria, was confirmed, but the mean of HDL cholesterol levels were 9.78% higher than the reference of World Health Organization.

**Conclusion:**

Although other factors might have contributed to lipid profile, the constant exposure to infection by *Plasmodium*, according to the physiology of the parasite, may have played an important role in defining the lipid parameters observed for this region. Further studies, such as the case–control is needed to confirm this hypothesis.

## Background

Malaria is recognized as a serious public health problem worldwide, affecting nearly 50% of the population in more than 109 countries. Its estimated 300 million new cases and 1 million deaths per year, mostly in children under 5 years and pregnant women in Africa [1]. The Amazon region is considered the country’s endemic area for malaria. In 2008 in Brazil, approximately 99% of malaria cases were concentrated in six states of the Amazon: Acre, Amapá, Amazonas, Pará, Rondônia and Roraima. This risk is measured by the annual parasite incidence (API), used to classify the areas of transmission in high, medium and low risk according to the number of cases per thousand inhabitants [2].

Cholesterol is obtained by means of cellular synthesis and about 70% is endogenous, from diet we have the exogenous cholesterol about 30% [3]. Studies have shown low levels of lipoproteins such as total cholesterol and your fractions, high density lipoprotein (HDL), and low density lipoprotein (LDL) in patients suffering of parasitic infection caused by *Plasmodium*[4]. In vitro studies have shown that independent of species, HDL cholesterol, is an essential source for the complete development of the malaria parasite (erythrocytic cycle) [5-7].

By the nature of cholesterol synthesis and physiology of the malaria parasite, the objective of this study were to assess the impact of malaria on lipid parameters of individuals living in an area considered hyperendemic in an association with the numbers of previous episodes of malaria.

## Materials and methods

The research site was the city of Anajás located in Pará, Brazil, Brazilian Amazon. Considered hyperendemic for the disease (API 57.7) situated in a microregion called Furo de Breves, mesoregion Marajó Island [8]. The study was a cross-sectional, the sample was chosen for convenience and approved by Committee of ethics in research with human of Instituto Evandro Chagas, protocol CEP/IEC – Nº 028/2010 CAAE: 0031.0.072.000-10. Were selected for the study 46 individuals, 31 male and 15 female, aged 20–60 years, without malaria but with a history of clinical disease and residents for more than five years in this city. It was established three groups according to the number of previous episodes of malaria: group I (n = 22) one to five episodes, group II (n = 20) six to ten episodes and group III (n = 4) eleven to fifteen episodes. Being measured the total cholesterol, HDL cholesterol and LDL cholesterol, from the serum samples collected of 10 ml of venous blood per individuals. For tests were employed commercial kits (ROCHE®) and clinical chemistry analyzer (COBAS INTEGRA 400®) following the manufacturer protocols and for the diagnosis of malaria disease was used the technique of thick smear [9].

## Results

**Figure 1:**
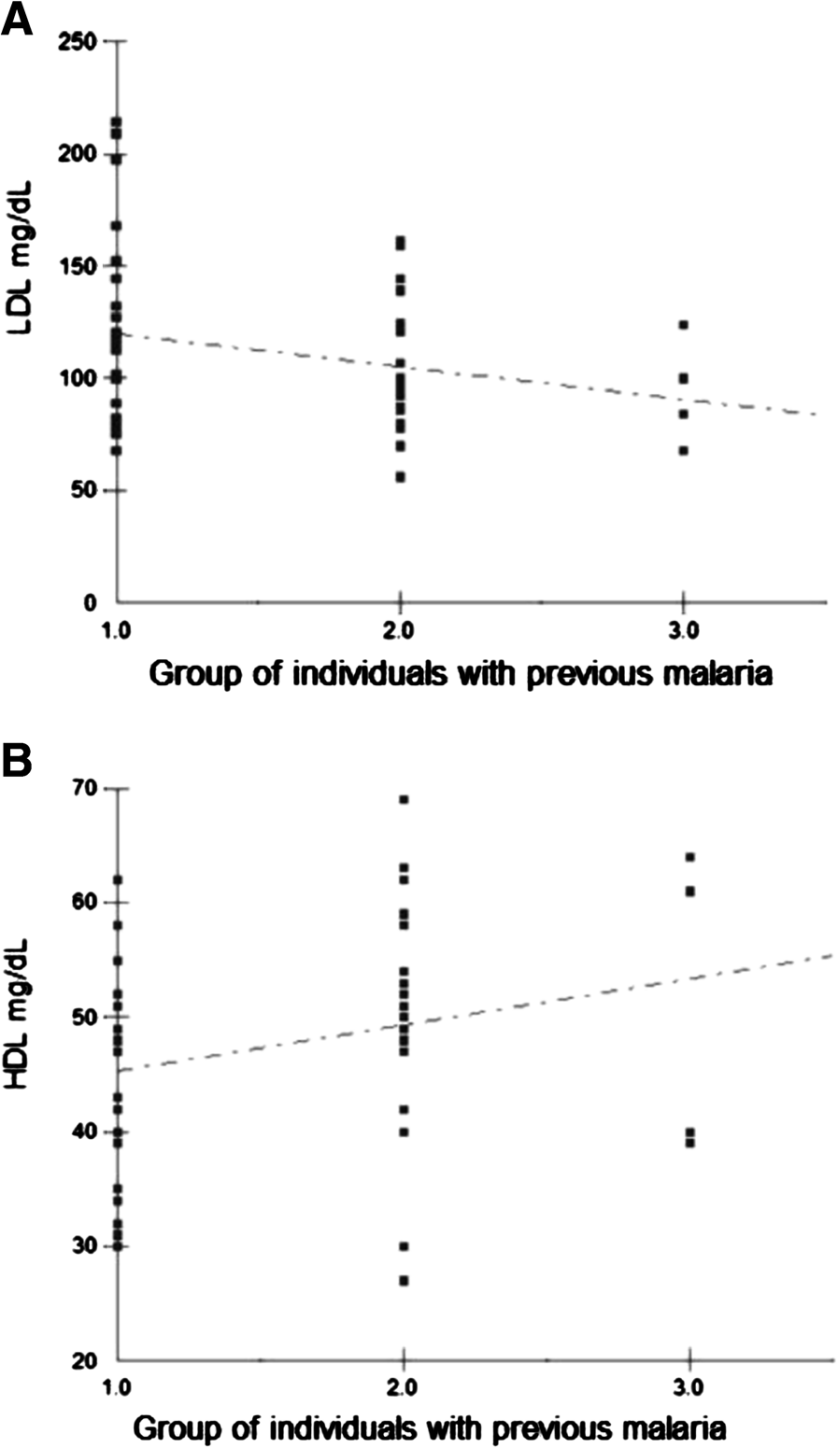
**Spearman linear correlation between HDL/LDL cholesterol levels and the number of previous episodes of malaria. (A)** A negative spearman linear correlation between LDL cholesterol levels and the number of previous episodes of malaria (r_s_) = − 0,194, p = 0,195. **(B)** A positive spearman linear correlation between HDL cholesterol levels and the number of previous episodes of malaria (r_s_) = 0,284, p = 0,055.

The mean of total cholesterol in the group I was 194.54 mg / dl, the group II of 181.90 mg / dl, and for the group III 154, 50 mg / dl. In LDL cholesterol, we found an average of 121.18 mg / dl for the group I, 103.70 mg / dl for the group II and 94.00 mg / dl for the group III. Analyzing the HDL cholesterol levels of patients according to the past of malaria, we found an average of 45.72 mg / dl for the group I, group II in the average was 51.00 mg / dl and the group III 51.35 mg / dl. As the analysis of the three groups of triglycerides, showed an average of 147.82 mg / dl for the first group of 149.55 mg / dl for the second group and 158.00 mg / dl for the third group as shown in Table 1. Using the Spearman linear correlation to HDL cholesterol with past malaria, we obtained a positive correlation (r_s_= 0.2838, p = 0.055), indicating that the more episodes of malaria, higher levels of HDL cholesterol in study subjects, and a negative correlation (r_s_ = - 0.1945, P = 0.1950) this time to LDL cholesterol, indicating that the more episodes of malaria smaller amount of LDL cholesterol in subjects of research, as shown in Figure 1.

**Table 1:** Distribution of values for the lipidic parameters and number of episodes of malaria for individuals the city of Anajás, Pará, Brazil, between 2011 and 2012

**Variables**	**group I**	**group II**	**group III**
	**(n = 22)**	**(n = 20)**	**(n = 4)**
*Total Cholesterol*			
Average	194.54 mg/dL	181.90 mg/dL	154.50 mg/dL
*Cholesterol HDL*			
Average	45.72 mg/dL	51.00 mg/dL	51.35 mg/dL
*Cholesterol LDL*			
Average	121.18 mg/dL	103.70 mg/dL	94.00 mg/dL
*Triglycerides*			
Average	147.82 mg/dL	149.55 mg/dL	158.00 mg/dL

## Conclusion

The hypocholesterolemia, the main characteristic of hyperendemic areas for malaria, was confirmed, but the mean HDL cholesterol levels were 9.78% higher than the reference of World Health Organization. Although other factors might have contributed to lipid profile, the constant exposure to infection by *Plasmodium*, according to the physiology of the parasite, may have played an important role in defining the lipid parameters observed for this region. Further studies, such as the case–control is needed to confirm this hypothesis.

## Abbreviations

API: Annual parasite incidence; HDL: High density lipoprotein; LDL: Low density lipoprotein.

## Competing interests

The authors declare that they have no competing interests.

## Authors’ contributions

FRN conceived the study, designed the study protocol and wrote the manuscript; RFL held clinical evaluation and performed the analysis and interpretation of data; AMV and MGF performed biochemical tests and exams thick smear and reviewed manuscript. All authors read and approved the final manuscript.
